# The haunted *Umwelt*: a biosemiotic and hauntological account of immune semiotic corruption in field cancerization

**DOI:** 10.1007/s12064-026-00496-5

**Published:** 2026-10-16

**Authors:** Anuraag Bukkuri

**Affiliations:** Department of Mathematics, City St George’s, University of London, London, UK

## Abstract

Field cancerization, the process wherein prior insults corrupt the semiotic landscape through which immune cells detect and respond to “the other”, persists even after the precipitating conditions resolve and the tissue histologically appears normal. This exemplifies a broader explanatory problem: How can we account for biomedical phenomena whose pathology is constituted by a history that standard mechanistic accounts cannot recover? I argue that a comprehensive understanding of field cancerization requires us to supplement molecular mechanistic explanations with lineage-level accounts, developed through a dialogue among Derrida’s hauntology, ghost criminology, and von Uexküll’s *Umwelt* as formalized through Peirce’s triadic model of signs. This framework generates a three-ghost taxonomy of immune semiotic corruption, explains why treating the present condition without addressing historically constituted corruption fails, and suggests concrete therapeutic implications based on this framework.

**Keywords**: field cancerization, immunology, biosemiotics, hauntology, ghost criminology, *Umwelt*, Derrida, Peirce

## Introduction

The idea of field cancerization (FC) was first proposed by Slaughter in 1953 to explain how epithelia that have been exposed to carcinogens acquire genetic and epigenetic alterations that can give rise to a multitude of independent, malignant lesions (Slaughter et al. 1953). Originally discovered in oral squamous cell carcinomas, FC has since been observed in nearly every epithelial tissue (Braakhuis et al. 2003; Dakubo et al. 2007). Despite considerable molecular characterization, we remain unsure why even complete clearance of malignant cells only provides transient benefits, often leading to rapid re-establishment of immunosuppression and high rates of local recurrence (Curtius et al. 2017; Dakubo et al. 2007; Li et al. 2024; Pierik et al. 2021; Tabor et al. 2001).

I suggest that this explanatory gap reflects a deeper conceptual limitation: the dogmatic emphasis on cancer as a disease of cellular identity (Slaughter 1944) rather than as a disease of communication and relational breakdown (Bukkuri and Adler 2021, 2024). Current accounts of FC are constitutive mechanistic explanations that aim to explain this higher-level phenomenon by detailing the properties of and interactions among lower-level molecular components (Krickel 2024; Machamer et al. 2000). Although these accounts are critical for fundamental biological understanding and for developing new drugs, they are temporally synchronic: their reference class for explaining biological phenomena is constrained to the current state of the system. However, FC is intrinsically diachronic: its essence is not merely mutated epithelia or the presence of aberrant molecular signals, but rather the persistence of a historically constituted, semiotically corrupted field whose pathological properties endure after the precipitating conditions resolve.

I argue that a comprehensive understanding of FC requires supplementing these mechanistic explanations with a lineage-level account (Calcott 2009) that examines how the immune semiotic landscape is corrupted through history, why this corruption persists, and how it reconstitutes even after standard interventions such as surgery. To develop this conceptual framework, I bring three philosophical ideas into dialogue: Jacques Derrida’s hauntology, which provides a philosophical grounding to understand how present conditions are inhabited by the specters of the past (Derrida 1994); ghost criminology, which applies and operationalizes hauntological thinking to environments that have historically been structured to perpetuate harm (Fiddler et al. 2024; Walklate 2023); and Jakob von Uexküll’s biosemiotic notion of the *Umwelt*, the unique self-centered sensory world experienced by a specific cell as manifested through Peirce’s triadic model, which allows us to understand how immune function is dysregulated not by intrinsic cellular defects, but by a corruption of the semiotic environment within which immune cells operate (Favareau 2009; Peirce 1934; von Uexküll 2010; Uexküll 1992).

These perspectives allow us to generate a three-ghost taxonomy of semiotic corruption, formalized via Peirce’s triadic model of signs (Peirce 1934). Ghost 1 corrupts the representamen, the information input to the immune cells. Ghost 2 corrupts the dynamic interpretant, the signal processing function within the immune cells that registers the representamen as “danger”. Ghost 3 corrupts the energetic interpretant, leading to a structural enforcement failure wherein immune cells are unable to kill malignant cells. The malignant cells that are the subject of much inquiry are not ghosts themselves but are the evolutionary consequences of these corrupted mechanisms. Under homeostatic environments, malignant cells are continually generated but are weeded out by the immune system. But under haunted conditions, the immune cells are ineffective at detecting and killing “dangerous objects”, thereby providing the malignant cells with a fitness advantage and facilitating their expansion, eventually culminating in a clinically detectable tumor.

The paper is structured as follows. In “Theoretical frameworks”, I ground the three theoretical frameworks outlined above in the context of FC. In “Hauntological synthesis: the three-ghost taxonomy”, I develop the hauntological synthesis, including the three-ghost taxonomy of semiotic corruption. In “Therapeutic implications: exorcising the three ghosts of semiotic corruption”, I provide implications of my framework for cancer treatment and map how current therapeutic efforts may help with exorcism of each of these ghosts.

## Theoretical frameworks

### Derrida’s hauntology

In his book *Specters of Marx*, Jacques Derrida introduced the idea of hauntology, a portmanteau of “haunting” and “ontology” that captures the idea that specters from the past haunt present conditions (Derrida 1994). Originally discussed in the context of Marxism haunting Western societies from beyond the grave, Derrida claims that specters operate in anachrony, i.e., that “time is out of joint”, allowing ghosts of the past to return and haunt the never-fully-self-contained present.

There are several aspects of Derrida’s hauntology that are relevant for my argument. First, hauntology specifies a temporal structure in which past events can persist as organizing forces into the present even as those events are unidentifiable in the current conditions. In the context of FC, this may be that the original carcinogenic insult has resolved and the tissue histologically looks normal, but the effects of the insults persist as corrupters of the semiotic landscape. These traces are not merely causal residues from the past that affect the present from “the outside” but are rather intimately embedded into the present identity. In other words, the tissue field is not just influenced by its carcinogenic history but is continually reconstituted by its traces on the semiotic landscape. Thus, effective therapies must not only address present conditions but also reconstitute the field’s identity via semiotic exorcism. Viewed through the lens of Caputo, building on Kierkegaard, such exorcisms are not backward-looking, aiming to erase the traces and recover a past that no longer exists (recollection). Rather, they are forward-looking, aiming to reorganize the current tissue into a configuration in which these traces can no longer serve as organizing forces towards malignancy (repetition) (Caputo 1987). It is in this sense that I will discuss exorcisms.

Another relevant aspect of hauntology concerns the future, the *arrivant* that the specter manifests: The ghosts of the past not only shape the present but affect future outcomes, i.e., the malignant transformation already exists within the milieu of the pre-malignant field. Critically, this nonlinear nature of time means that blocking the transition from pre-malignancy to malignancy is as much a problem of cancer treatment and reversion as it is a problem of cancer prevention. In other words, the distinction between cancer prevention and cancer treatment efforts is not a meaningful ontological one, but rather a pragmatically useful epistemological one, capturing two observational positions on the same underlying condition.

### Ghost criminology

Ghost criminology, which deals with environments that have historically been structured to perpetuate harm (Fiddler et al. 2024; Walklate 2023), allows us to further apply and operationalize notions of haunting to FC by shifting the explanatory level from cells to the tissue environment. Although developed in the context of sociology, the underlying concepts are transferable to our analysis of FC. Namely, criminogenic environments and pre-malignant fields are constituted by historical processes rather than solely by actors in the present state and are characterized by the corruption of internal control mechanisms (e.g., degrading of signaling integrity) through these processes. Pathology in both cases arises not from individual bad actors but from the environmental-level properties that these historical processes gave rise to. Cancerous tissues are thus not just tissues containing cancerous cells but are rather environments that have been systematically corrupted over time to undermine mechanisms of cellular social control. Not only does such a tissue systematically disadvantage non-cancerous cells, but it also corrupts the mechanisms of tissue resilience that would normally allow these cells to recover and re-establish homeostasis. For this reason, cancer is often conceptualized as a “wound that never heals” (Byun and Gardner 2013; Dvorak 2015; Hua and Bergers 2019; Virchow 1858). Viewed through Peirce’s triadic model (Peirce 1934), this means that the tumor is not the object as is commonly believed, but is rather merely the representamen. As stressed before, therapies that solely attempt to remove the representamen and do nothing to treat the underlying object will remain limited in efficacy^1^.

### The biosemiotic umwelt

**Fig. 1:**
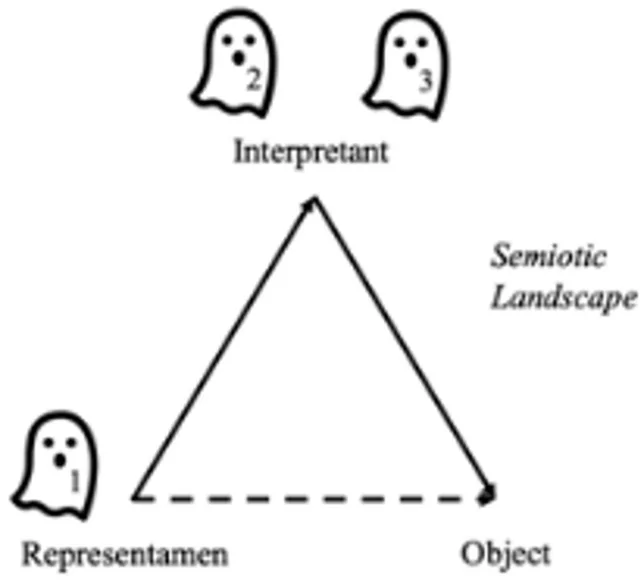
Triadic model of signs with ghosts corresponding to corrupted aspects of the cancer-immune semiotic landscape

Jakob von Uexküll’s *Umwelt* captures the sensory world as experienced by each individual agent: individuals do not experience an objective environment, but rather a unique perceptual world defined by the representations of the objects they encounter and their interpretations of them (von Uexküll 2010; Uexküll 1992). This notion can be formalized using Peirce’s triadic model of signs, in which a sign is a dynamic three-part relationship composed of an object, a representamen, and an interpretant (Peirce 1934) (Fig. 1).

The object captures what the sign refers to, the representamen is the form that the sign takes, and the interpretant determines the meaning of the sign. It is worth noting that Peirce’s theory of signs is much richer than I have described, encompassing multiple sign types (e.g., icons, indices, symbols) and interpretants (e.g., immediate, dynamical, final, energetic, logical, acting as ground, in mind, and in quasi-mind). In this paper, I specifically focus on those aspects that are most relevant to immune semiotic corruption.

Of particular relevance, Peirce distinguishes between two kinds of interpretants: the dynamic and the energetic. The former is concerned with the actual effect that the sign produces “in the mind”, whereas the latter deals with the action that the sign induces. For instance, consider the case of a fire in a kitchen: the fire-as-signified is the object, the smoke alarm ringing is the representamen, the dynamic interpretant is the understanding that something is burning, and the energetic interpretant is evacuating the building. The idea of *Umwelt* means that the unique experience of individuals is not only due to differences in the representamen that they encounter (closely related to the idea of occupying distinct ecological niches (Holt 2009), but in their interpretations of these signs. Although the *Umwelt* concept was originally developed in the context of human and animal perception, its application to agents without nervous systems, such as cells, is well established in the biosemiotics literature: the key is not cognition, but rather the ability to distinguish and respond differently to distinct representamen (Barbieri 2007; Favareau 2009; Hoffmeyer 2008; Kull 1999).

As I will discuss in the next section, corruption of the cancer-immune semiotic system can occur in one of three ways, corresponding to distinct parts of the Peircean triad (Fig. 1):

1. Representamen falsification: the object is dangerous but the representamen has been falsified to communicate normalcy to the immune cells (analogous to silencing of the smoke alarm).
2. Dynamic interpretant corruption: the immune cells read the danger signal correctly, but its meaning has been corrupted (analogous to residents being trained to ignore the fire alarm).
3. Energetic interpretant inhibition: the immune cells recognize the danger of the object, but the translation from interpretation into action has been inhibited (analogous to fire exits being blocked).

## Hauntological synthesis: the three-ghost taxonomy

Derrida’s hauntology, ghost criminology, and von Uexküll’s *Umwelt* converge on the claim that the immune semiotic landscape is corrupted at three levels, each corresponding to a distinct aspect of the Peircean triad. I call these the three ghosts of FC. Although biosemiotics has examined the corruption of the semiotic structure in various contexts (Hoffmeyer 2008), the application to oncogenesis and the specific three-ghost taxonomy developed here are novel.

### Ghost 1: representamen falsification

Ghost 1 is concerned with representamen falsification, in which dangerous objects mask their identity to immune cells. These immune cells accurately read and interpret the signals of the malignant cells, but the signals themselves are corrupted. Biologically, this occurs via field-wide epigenetic silencing of immune recognition machinery. For instance, MHC-1 molecules present neoantigens on the cell’s surface that allow cytotoxic CD8 + T cells to detect and kill cancer cells. However, tumors often downregulate MHC-1 expression to evade immune recognition (Dhatchinamoorthy et al. 2021; Kong et al. 2025; Wu et al. 2023). Similarly, NKG2D ligands are stress-induced proteins (MICA/B, ULBP1-6) normally expressed on malignant cells that are detected and marked by natural killer cells and CD8 + T cells for killing. However, cancer cells can shed these ligands from their cell surface using ADAM/MMP metalloproteases and evade immune detection (Dhar and Wu 2018; Huang et al. 2024; Tan et al. 2023). The hauntological structure of Ghost 1 is encoded in the malignant cell itself: Although the carcinogenic insult that initially drove silencing of immune presentation machinery has subsided, the epigenetic mark remains on the cell’s surface—the cell does not “remember” its history, but this Derridean trace shapes its present identity.

### Ghost 2: dynamic interpretant corruption

Ghost 2 is concerned with corruption of the dynamic interpretant, the signal processing system of immune cells that allows them to register malignant signals as “danger”. Even if the representamen faithfully captures the malignant nature of the object, the immune system’s interpretative response may be pathologically reprogrammed to interpret such signals as benign. For instance, persistent viral infection drives sustained immune activation that gradually recalibrates the immune system towards tolerance via modification of internal cellular machinery and the local microenvironment to promote immune suppression (Medzhitov et al. 2012; Trinchieri 2012). Although this is an evolutionary conserved response to avoid autoimmunity, it leads to ignorance of malignant cells as what may have previously been interpreted as a danger signal is now viewed as merely background noise. Indeed, epidemiological studies link chronic hepatitis B and C infection to hepatocellular carcinoma (Abdelhamed and El-Kassas 2024; Awadh et al. 2024; Huang et al. 2011; Wang et al. 2025), *H. pylori* infection to gastric cancer (Duan et al. 2025; Wroblewski et al. 2010), and HPV infection to cervical cancer (Burd 2003; Choi et al. 2023; Okunade 2020). The hauntological structure of Ghost 2, a paradigmatic example of Roland Barthes’ myth (Barthes et al. 2006; Barthes and Barthes 1977), leaves its mark on the immune interpretant: even after the chronic disease is resolved, the signal processing system of the immune system remains corrupted and biases interactions with subsequent malignant actors towards tolerance. In Peircean terms, this chain of semiosis is realized in an ultimate logical interpretant: a disposition to interpret future signals in a particular manner (Peirce 1934; Rosenthal 1990). The corruption of the dynamic interpretant leads to this *habit*, which outlives the pathological conditions that precipitated it.

### Ghost 3: energetic interpretant inhibition

Even if immune cells recognize and interpret malignant cells as dangerous objects that must be eliminated, their execution may be corrupted at the physical and molecular levels by the effects of Ghost 3. Physically, stiffening of the extracellular matrix (ECM) often results from collagen cross-linking and fibrosis associated with repeated tissue injuries; this inhibits immune infiltration into the tumor (Horn et al. 2022; Hu et al. 2025; Nicolas-Boluda et al. 2021; Sun et al. 2021). If immune cells are able to infiltrate the tumor, they may interact with molecules that inhibit their effector functions. They may encounter TGF-β, an immunosuppressive cytokine that suppresses cytotoxic T lymphocytes via inhibition of perforin, granzyme A, granzyme B, Fas ligands, and interferon gamma, collectively responsible for the cytotoxic properties of these cells (Thomas and Massagué 2005). Similarly, they may experience reduced levels of L-arginine, an essential amino acid for T lymphocyte function, due to increased metabolism by myeloid derived suppressor cells (Rodríguez and Ochoa 2008). The hauntological structure of Ghost 3 is the most concrete, both literally and figuratively: the material properties of the ECM, for example, were established during prior periods of insult and inflammation but may persist long after the injury is resolved.

## Therapeutic implications: exorcising the three ghosts of semiotic corruption

With an understanding of how immune semiotic corruption occurs and persists, I now discuss the therapeutic implications of this framework. Under tissue homeostasis, malignant cells are identified (faithful representamen), interpreted (accurate dynamic interpretant), and eliminated from the population (effective energetic interpretant). However, when the immune semiotic landscape is corrupted, these cellular social controls break down. This confers malignant cells with a fitness advantage, allowing them to expand in the tissue. Critically, these cells are not ghosts themselves but are rather the downstream consequences of haunted conditions. Most current therapies such as surgery focus on directly killing these malignant cells; however, these efforts will have limited, transient impact as the ghosts that drive semiotic corruption have not been exorcised and thus, the conditions that produce malignancy remain. As viewed through Derrida’s notion of time being “out of joint”, a reconstitution of the immune semiotic landscape (a repetition rather than a recollection, *sensu* Caputo (Caputo 1987) is required to reverse the predisposition to cancer (the malignant *arrivant*) that is present in the haunted tissue. To this end, I will briefly discuss current therapeutic efforts that aim at exorcising the ghosts outlined in  “Hauntological synthesis: the three-ghost taxonomy” .

Exorcising Ghost 1 requires restoring representamen fidelity. Histone deacetylase inhibitors, small molecule inhibitors, and DNA methyltransferase (DNMT) inhibitors have all shown promise at reversing epigenetic silencing of antigen presentation machinery via upregulation of NKG2D ligands and MHC-I receptors, improving the accuracy of what malignant cells present to the immune system (Armeanu et al. 2005; Poggi et al. 2009; Schmiedel and Mandelboim 2018; Shanmugam et al. 2022; Sun et al. 2019; Ullrich et al. 2013).

Exorcising Ghost 2, a feat that has thus far proved challenging, requires restoring the dynamic interpretant. Most efforts to this end concentrate on addressing the chronic condition from which the ghost arose by using, e.g., antiviral or anti-inflammatory medications. Although this can remove the current molecular factors that actively promote and exacerbate interpretant corruption, it does not directly restore interpretant fidelity. This notion is supported by clinical observations, in which treatment of hepatitis C with direct-acting antivirals reduced hepatocellular carcinoma risk, though not to baseline levels (Kanwal et al. 2017). Another approach involves using the DNMT inhibitors described above: these drugs not only improve antigen presentation on malignant cells, but also restore expression of inflammatory response genes in immune cells, combating the tolerance bias associated with immune interpretant corruption (Chiappinelli et al. 2015). Therapeutic vaccines that re-educate immune cells to interpret tumor cells as danger instead of background noise by presenting tumor-associated antigens in strongly immunogenic contexts are also currently under investigation, and have showed some promise in treating melanoma (Ott et al. 2017; Sahin et al. 2017).

Exorcising Ghost 3 requires restoring the enforcement capacity for the energetic interpretant. To this end, efforts have mainly focused on anti-fibrotic therapies and ECM-modifiers that target cancer-associated fibroblasts, myeloid derived suppressor cells, the TGF-β pathway, and ECM collagen cross-linking (Hauge and Rofstad 2020; Huang et al. 2021; Jou et al. 2024; Lee et al. 2025; Van Den Bulk et al. 2021). These therapies remodel the microenvironment to remove the physical and molecular factors that sever the effector-interpretant relationship. Note that the pharmakon (Derrida 1983) rears its head here: many commonly-used treatments such as surgery, chemotherapy, and radiation therapy can themselves induce tissue damage and fibrosis (Bukkuri 2026; Mancini and Sonis 2014), seeding the trace that constitutes Ghost 3—contending with the inherently dual nature of interventions will be critical to designing holistic treatment strategies for cancer.

It is important to recognize that durable treatment responses require exorcism of all three ghosts. As Peirce suggests, the sign is a manifestation of the dynamic interplay among object, representamen, and interpretant (Peirce 1934)—corruption in any of these factors, or their relations, is bound to produce a corrupted sign. Thus, therapeutic efforts that aim at anything less than the exorcism of all three ghosts are bound to have limited efficacy.

## Conclusion

Field cancerization is an inherently hauntological phenomenon—it is a tissue microenvironment that contains traces of prior insults, injuries, and carcinogenic events. These specters are not just causal residues from “the outside” but rather persist as organizing forces in the current environment, corrupting the semiotic landscape within which immune cells sense, interpret, and respond to danger, and become an integral component of the present tissue’s identity. In this paper, I introduced a three-ghost taxonomy to capture the main sources of corruption in the context of cancer: representamen falsification, dynamic interpretant corruption, and energetic interpretant inhibition. In doing so, I have provided a formal vocabulary for distinguishing among three types of semiotic corruption and for identifying specific targets for therapeutic intervention. Treatment strategies such as surgery that do not exorcise all three ghosts are bound to have limited treatment efficacy (Bukkuri 2026). To this end, I propose the administration of combination therapies that restore antigen presentation machinery on malignant cells, re-educate immune cells to interpret malignant signals as danger instead of background noise, and remove physical and molecular barriers to promote effective implementation of malignant cell killing. To prevent malignant transformation in the present requires us to first fix the injustices of the past: to exorcise the ghosts that linger from prior insults before their malignant *arrivant* is manifested, and to prevent the injuries that generate the semiotic-corrupting traces in the first place.

## Data Availability

No datasets were generated or analysed during the current study.

## Footnotes

1 This suggests a larger problem of using biomarkers to guide clinical decision making: Biomarkers are representamen, not objects. Thus, evolutionary processes may operate to decouple the signal reliability of biomarkers, thus degrading their utility as disease markers (Bukkuri and Adler 2023; Strimbu and Tavel 2010).
